# Variations in tissue optical parameters with the incident power of an infrared laser

**DOI:** 10.1371/journal.pone.0263164

**Published:** 2022-01-31

**Authors:** Omnia Hamdy, Haitham S. Mohammed

**Affiliations:** 1 Engineering Applications of Lasers Department, National Institute of Laser Enhanced Sciences, Cairo University, Giza, Egypt; 2 Biophysics Department, Faculty of Science, Cairo University, Giza, Egypt; Universiti Brunei Darussalam, BRUNEI DARUSSALAM

## Abstract

Infrared (IR) lasers are extensively utilized as an effective tool in many medical practices. Nevertheless, light penetration into the inspected tissue, which is highly affected by tissue optical properties, is a crucial factor for successful optical procedures. Although the optical properties are highly wavelength-dependent, they can be affected by the power of the incident laser. The present study demonstrates a considerable change in the scattering and absorption coefficients as a result of varying the incident laser power probing into biological samples at a constant laser wavelength (808 nm). The optical parameters were investigated using an integrating sphere and Kubelka-Munk model. Additionally, fluence distribution at the sample’s surface was modeled using COMSOL-multiphysics software. The experimental results were validated using Receiver Operating Characteristic (ROC) curves and Monte-Carlo simulation. The results showed that tissue scattering coefficient decreases as the incident laser power increases while the absorption coefficient experienced a slight change. Moreover, the penetration depth increases with the optical parameters. The reduction in the scattering coefficients leads to wider and more diffusive fluence rate distribution at the tissue surface. The simulation results showed a good agreement with the experimental data and revealed that tissue anisotropy may be responsible for this scattering reduction. The present findings could be considered in order for the specialists to accurately specify the laser optical dose in various biomedical applications.

## Introduction

Nowadays, optical methods are widely utilized in medical diagnosis and treatment due to their high safety and functionality [1–3]. In typical procedures, light in the red to the near-infrared range is used to probe the tissue while the reflected/transmitted light is collected using detectors or CCD cameras. The captured reflected/transmitted light provide important information about tissue pathology [4]. The light reflection and transmission by the tissue are diffused in nature due to the inhomogeneity and dense scattering characteristics of biological tissue [5].

The propagation of light in the tissue is affected by the tissue absorption and scattering parameters. These parameters are wavelength-dependent and specific for each tissue type [6]. The optical dose is greatly affected by the scattering and absorption parameters of the examined tissue; therefore, an accurate calculation of these parameters is highly required. Tissue optical parameters can be determined using different mathematical models that are based on the light radiative transfer equation (RTE) and diffusion theory of light transport [6]. Moreover, some numerical methods such as Monte-Carlo (MCML) and inverse adding doubling (IAD) can be used for the same purpose [7]. However, utilizing most of the previously mentioned mathematical methods for determining tissue’s absorption and scattering characteristics requires experimental data of the tissue’s optical reflectance and/or transmittance [8, 9]. These experimental data can be obtained by using either integrating spheres [10–12] or distant detectors [13–15].

It should be highlighted that the most relevant studies in literature investigated the dependence of the tissue optical parameters on the wavelength of the incident light without considering the incident laser power [16–21]. However, other researches demonstrated the effect of the temperature-dependent optical properties on the fluence and temperature distribution within some biological tissues during low-level laser therapy [22]. It was reported that the photo-thermal effect of the utilized laser source, such as heating and coagulation, can change the tissue optical properties [8, 23]. In case of implementing low-level laser beam (i.e., from 50 to 500 mW) in therapeutic application, it is of paramount importance to study the variations in tissue optical parameters as a function of laser beam characteristics in order to optimize the therapeutic outcomes [24].

The variation of scattering properties of the biological samples with laser intensity (or fluence) has not been yet investigated thoroughly. A controlled study that tackles this subject is required. Furthermore, the present investigation may gain more attention in biomedical applications by taking into consideration the inhomogeneity of the biological samples and how this can greatly affect the scattering of laser in the tissue. The present study aimed to calculate the optical scattering and absorption coefficients of samples of rat’s skull and skin at different incident laser power (150, 200, 225, 250, 300, and 350 mW). The optical parameters are determined using the Kubelka-Munk mathematical model based on experimentally measured diffused light. A single integrating sphere-based optical setup is used to collect experimental data. The penetration depth and spatial fluence rate distribution at the surface of the examined samples are estimated. In addition, the numerical simulation is validated by the experimental results.

## Materials and methods

The protocol of measurement can be described as follows; after sample preparation, the sample is placed in the integrating sphere for measuring transmittance and reflectance. The experimental data are saved and analyzed via spectrometer software. The collected data are then processed using the mathematical model (Kubelka–Munk) in order to estimate the optical parameters. The obtained optical parameters are introduced to the diffusion equation in order to obtain the optical fluence distribution. The average and root mean square (rms) values of five consecutive measurements of the same sample are used for visualizing the results. Such procedure is repeated at different incident laser powers. Furthermore, the obtained results are verified using ROC and Monte-Carlo simulation methods.

### Samples preparation

Skull and skin samples taken from six male Wistar rats weighing from 180 to 200 g were investigated in the present study. A total of 16 samples (6 skull samples and 10 skin samples) were used for each measurement 5 replicates were done). The experimental animals were obtained from the breeding center at the National Research Center (NRC) in Egypt. All the experimental procedure followed the international guidelines of animal care and approved by the committee of institutional animal care and use at Cairo University (No. CU/I/F/46/19). Under an anesthetic dose (Sodium pentobarbital injected intraperitoneal of a concentration of 40 mg/kg as anesthetic drug), animals were decapitated when skull and skin samples were dissected. All skin samples were cut from the same location from the animal’s dorsal skin, shaved by an electrical trimmer and razor blade, and cleaned with an alcohol swap. Skull samples were cut from the animal’s head which is followed by its skin removal. The skull samples were immersed in hydrogen peroxide (H_2_O_2_) for a complete removal of skin tissue debris. The Freshly prepared skin samples were cut into 2.5 cm in length and 3.5 cm in width to fit into the integrating sphere holder. The thickness of the examined samples was determined via a fine Vernier caliper and a micrometer. In the present study, skull and skin tissues were used to represent both hard and soft biological tissues, respectively.

### The optical setup

**Fig 1:**
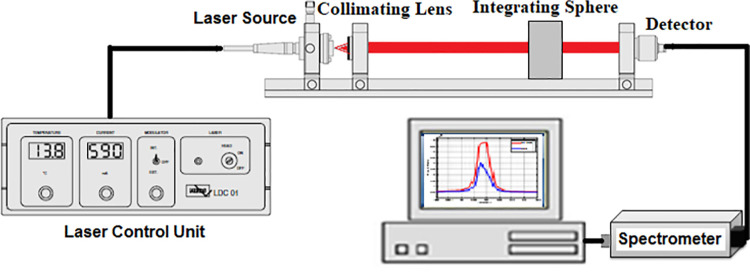
Schematic depiction of the experimental laser.

In the present work, a power controllable semiconductor laser diode of a wavelength of 808 nm and a maximum output power of 500 mW was utilized. The laser output is controlled by the temperature and the injection current of the laser control unit (LDC01, MEOS GmbH- Germany) as illustrated in Fig 1. It is noteworthy to mention that the irradiance is kept constant for all measurements. Moreover, the samples’ surface temperature during the experiments is monitored using a digital temperature controller and a thermocouple (XH-W3001, Generic, China). However, such low radiation power has almost no thermal effect for relatively short exposure time. A plano-convex lens (mention the specs) is used to collimate the output beam to the detector. The detector is connected to a compact spectrometer (STDFSM, Touptek Photonics Co. Ltd- China) via optical fiber cable. The collected signal is analyzed using the spectrometer and in-house developed Matlab code.

**Fig 2:**
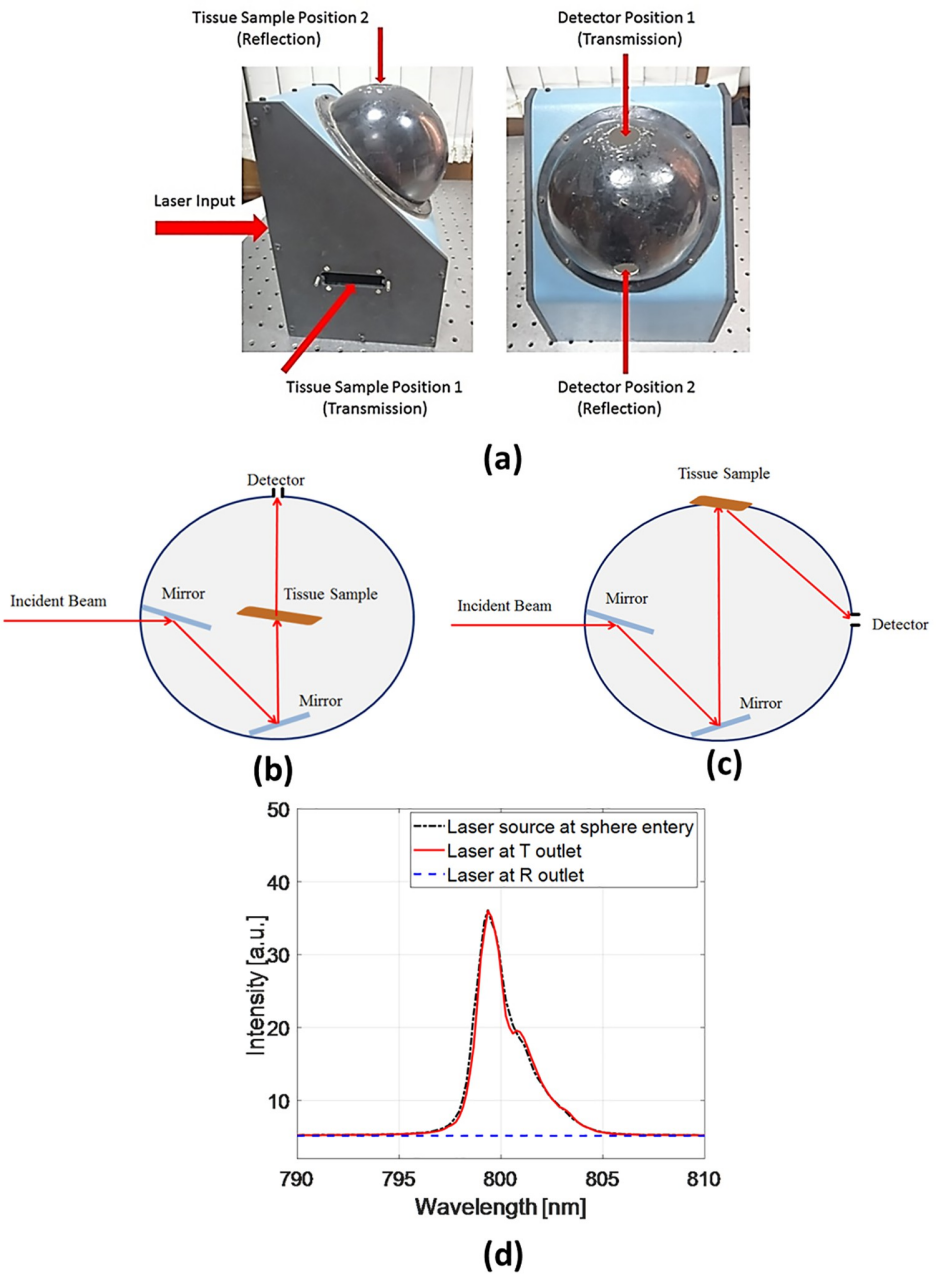
Integrating sphere configurations (a) positions for incident laser, sample, and detector, (b) transmission measurement, (c) reflection measurement, and (d) transmitted and reflected signals without sample.

The optical properties of the skull have been calculated using the Kubelka-Munk mathematical method [25]. It is a transport model that estimates the absorption and reduced scattering coefficients of the examined tissue using the experimentally measured diffuse reflectance and transmittance [9, 26]. In the present study, the diffuse reflectance R_d_ and transmittance T_d_ of the selected samples have been measured using a single integrating sphere-based optical setup. The integrating sphere contains two positions for the sample; position A and position B for measuring the reflectance and transmittance, respectively. The output signal is detected using a CCD detector (TCD1304AP) which is connected to STDFSM digital fiber spectrometer through a data collecting optical fiber. Finally, a computer is employed for data analysis and processing as illustrated in Fig 2.

In the transmission mode (Fig 2B), the sample is placed inside the sphere, then the incident laser beam probes the sample after being directed by two mirrors and, hence, the detector records the total transmitted light. Fig 2C illustrates the reflection mode where the sample is placed at the upper port of the sphere while the input laser is directed to the sample using the two mirrors. Then, the sample’s diffuse reflectance collected by the sphere is captured by the detector. To ensure that the light radiation is delivered without significant losses inside the integrating sphere, the transmitted and reflected signals were measured before inserting the biological sample inside the sphere (see Fig 2(D)). Such measurements are performed as a system calibration. It is supposed that in an empty integrating sphere, there is no detected diffuse reflectance and the incident laser beam will be totally recorded as a transmittance. Therefore, as demonstrated in Fig 2(D), the laser intensity at the sphere entry is almost equal to that transmitted to the sphere outlet (T) whereas there is no reflected intensity (R) (demonstrated by the horizontal dashed line) since the sample to be measured is not placed.

### Calculating the absorption and scattering coefficients

Kubelka–Munk model can be applied to estimate tissue absorption and reduced scattering coefficients with the approximation that the radiance is diffuse only [5]. This model assumes two diffuse fluxes inside the tissue; one in the same direction of the incident light and another one is the backscattered flux in the opposite direction [27]. Two Kubelka–Munk coefficients A_KM_ and S_KM_ are assumed for absorption and scattering of diffuse radiation, respectively, and can be presented in terms of the experimentally measured values of diffuse reflectance and transmission, as follows:

$$R_{d}=\frac{\sinh\;S_{KM}YD}{X\;\cosh\;S_{KM}YD+Y\;\sinh\;S_{KM}YD}$$ (1)

$$T_{d}=\frac{Y}{X\;\cosh\;S_{KM}YD+Y\;\sinh\;S_{KM}YD}$$ (2)

where D is the optical thickness of the slab to be considered, and the parameters X and Y can be expressed as:

$$X=\frac{1+R_{d}^{2}-T_{d}^{2}}{2R_{d}}$$ (3)

$$Y=\sqrt{X^{2}-1}$$ (4)

According to Kottler et al. [28] the convenience of applying the Kubelka–Munk method arises from the fact that scattering and absorption coefficients can be directly calculated from measured reflection and transmission coefficients as:

$$S_{KM}=\frac{1}{YD}\ln\left[\frac{1-R_{d}(X-Y)}{T_{d}}\right]$$ (5)

$$A_{KM}=(X-1)S_{KM}$$ (6)

Then, the relation between S_KM_ and A_KM_, and the absorption and reduced scattering coefficients (*μ*_*a*_ and *μ*_*s*_′) of the sample can be expressed as:

$$A_{KM}=2\mu_{a}\;and\;S_{KM}=\frac{3}{4}\mu_{s}\prime -\frac{1}{4}\mu_{a}$$ (7)

From Kubelka-Munk calculations, absorption and reduced scattering coefficient are obtained. The light penetration depth δ can be calculated using the absorption and reduced scattering coefficients as follows [29]:

$$\delta =\frac{1}{\sqrt{3\mu_{a}(\mu_{a}+\mu_{s}^{\prime })}}$$ (8)

Such parameter is very important for the correct calculation of the optical dose in many medical procedures such as photodynamic therapy [30].

### Simulating fluence rate at the sample surface

The spatial distribution of the fluence rate at the surface of the skull and skin samples has been determined using the finite element solution of the diffusion equation [13, 14]. The steady-state diffusion Eq (9) can be modeled by the Helmholtz Eq (10) under the environment of COMSOL Multiphysics software, as follows:

$$\frac{\partial \phi (\vec{r},t)}{c\partial t}+\mu_{a}\phi (\vec{r},t)-\nabla_{\cdot }[D\nabla \phi (\vec{r},t)]=S(\vec{r},t)$$ (9)

**Fig 3:**
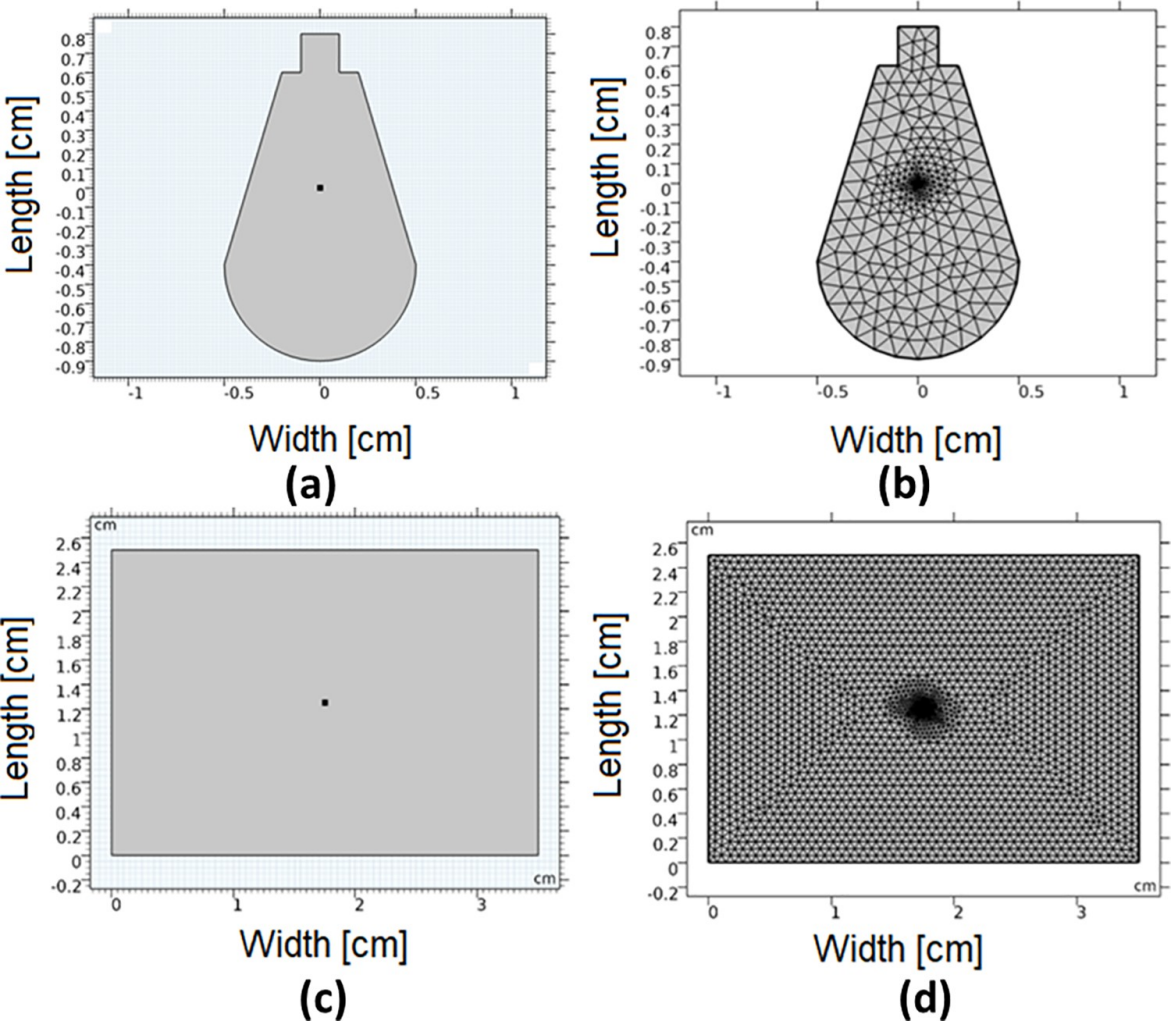
The finite element geometry (a) model for skull, (b) mesh for skull, (c) model for skin, (d) mesh for skin.

∇(−C∇u)+au=f (10)

where the constant $D=\frac{1}{3(\mu_{a}+\mu^{\prime }{}_{s})}$ is the diffusion coefficient, *μ*′_*s*_ = (1−*g*)*μ*_*s*_ is the reduced scattering coefficient, and g is the anisotropy factor. $S(\vec{r},t)$ represents the source term and $\phi (\vec{r},t)$ is the fluence rate. Comparing the parameters in 8 with those in 9 we get *u* = *ϕ*, *C* = *D*, *a* = *μ*_*a*_, and *f* = *S*. The implemented finite element model and its corresponding mesh for both skull and skin samples are presented in Fig 3. The point in the middle of the model simulates the laser source.

### Receiver Operating Characteristic (ROC)

Receiver operating characteristic (ROC) is a graphical representation that is used to evaluate the accuracy of diagnostic tests by plotting the sensitivity versus 1-specifity of a studied test or a combination of tests [31, 32]. The area under the ROC curve (AUC) is considered the key factor that assesses the efficiency of the studied discrimination method (test) as the more accurate test the greater AUC [33, 34]. For two random variables X and Y of distribution functions F and G, respectively, at any point p, the sensitivity of the test is defined as Se(p) = 1 − G(p) while the specificity is given by Sp(p) = F(p). Thus, plotting Se(p) versus 1−Sp(p) for -∞≤p≤∞ represents the ROC curve [35].


$$ROC(t)=1-G(F^{-1}(1-t)),over\;t\in [0,1]$$ (11)

$$AUC=\int_{0}^{1}ROC(u)du$$ (12)

It should be noted that creating a ROC curve is based on the true positive, true negative, false positive, and false negative concept. The X-axis of the curve represents 1- specifity which is the false positive fraction whereas the Y-axis shows the sensitivity which demonstrates the true positive fraction [36]. Our presented ROC curves have been created under the environment of the MATLAB software package.

### Monte-Carlo simulation

In the present study, Monte-Carlo simulation has been applied for results verification. Monte-Carlo simulation is a very common numerical method used to simulate light propagation in single and/or multi-layers turbid media [37–39]. Refractive index, absorption coefficient, scattering coefficient, anisotropy, and sample thickness are the five tissue-related parameters required to run the simulation code named “MCML” [40]. The output of the simulation can give sufficient information about tissue diffuse reflectance, fluence rate, and absorbed fraction [41, 42].

## Results

### Dependence of optical parameters on the incident laser power

**Fig 4:**
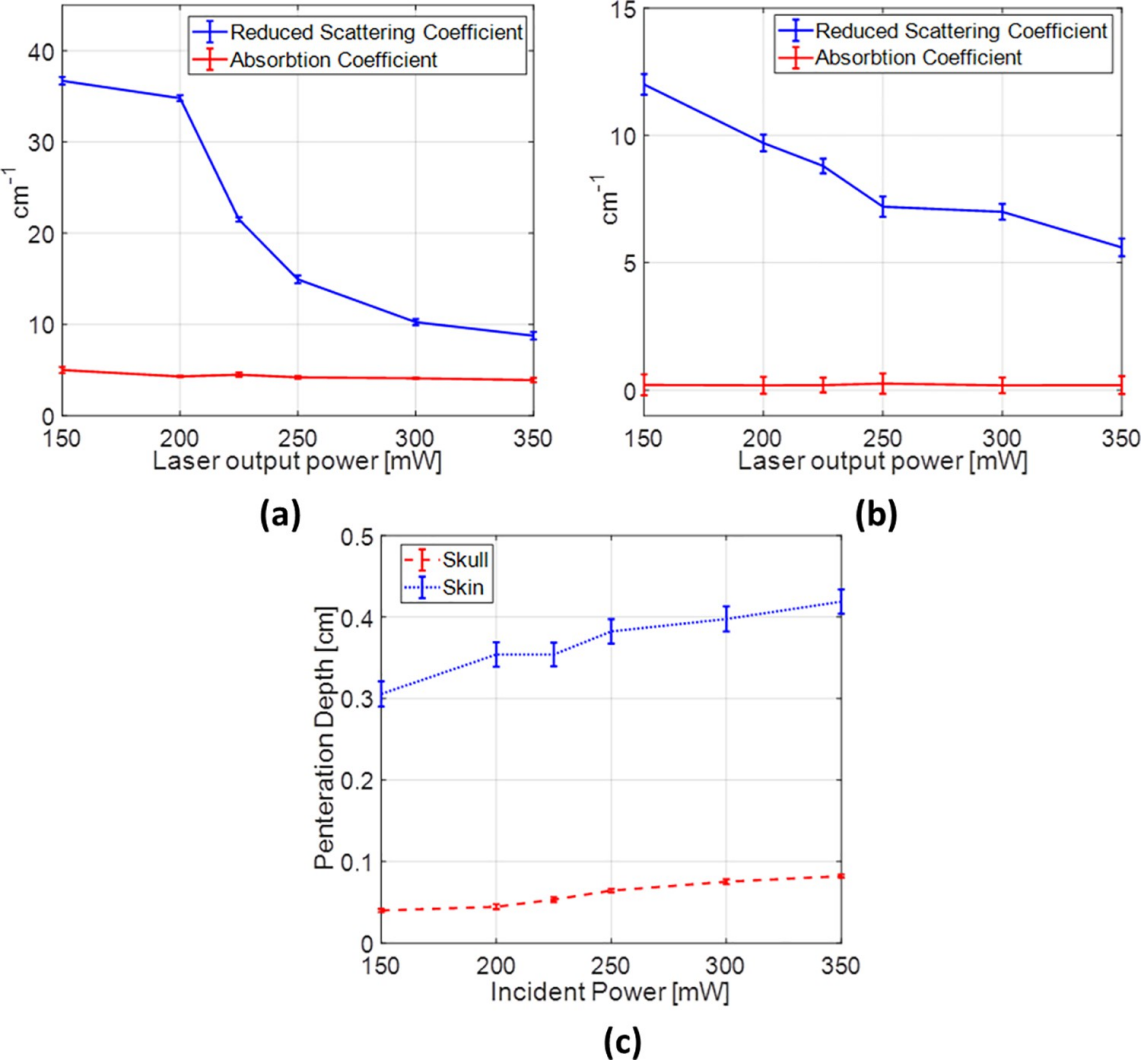
Variation of the absorption coefficient and the reduced scattering coefficient with the laser power, (a) skull, (b) skin, (c) variation in the penetration depth for the skin and skull samples.

The experimental data of reflectance and transmittance revealed that the total transmittance increases by increasing the incident power. On the other hand, the diffuse reflectance R_d_ decreases at higher incident laser power. The absorption and reduced scattering coefficients of the skull and skin samples have been calculated at different incident laser powers. The variation in their values is presented in Fig 4A, and 4B. The variation in the optical penetration depth versus the laser incident power is illustrated in Fig 4(C).

As illustrated in Fig 4A and 4B, the reduced scattering coefficient decreases by more than 50% and 30% in the skull and skin, respectively, as the incident laser power increases. On the other hand, there is almost no change in the absorption coefficient for both examined tissues. In both cases, the penetration depth increases by increasing the incident power as a result of the new combination of the absorption and scattering coefficient.

### Verification using ROC curves

**Fig 5:**
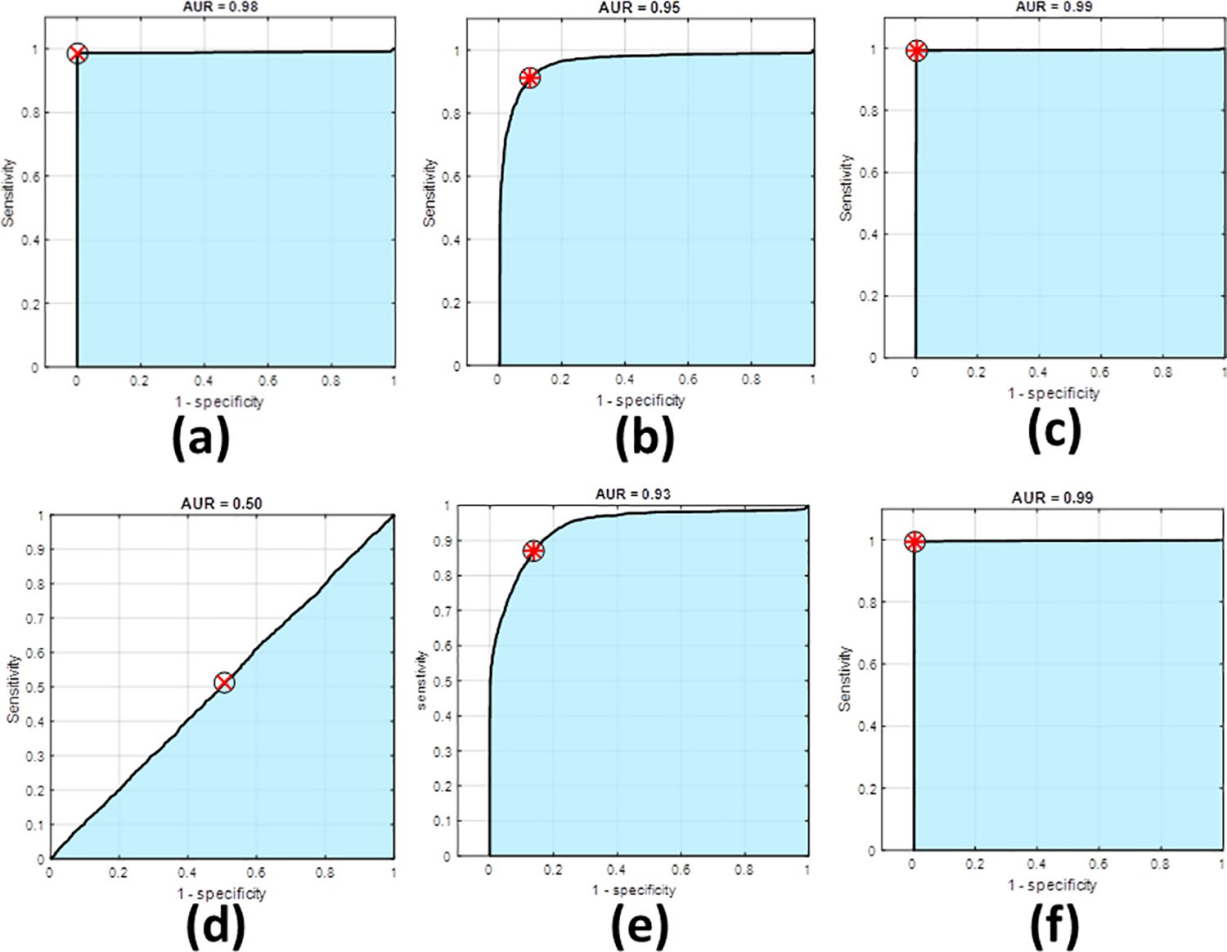
ROC curves of diffuse transmittance in skull (a) between 150 and 200mW, (b) between 200 and 225mW, (c) between 225 and 250 mW, (d) between 250 and 300 (e) between 300 and 350 mW, (f) between 150 and 350 mW.

For evaluating the accuracy of the obtained results, ROC curves of the experimental data have been constructed. ROC curves of the measured diffuse transmittance spectra at each two consecutive incident powers have been plotted for both skull and skin samples as presented in Fig 5, respectively.

As shown in Fig 5, the between 200 and 225 mW, and 225 and 250 mW. However, lower discrimination appears between 150 and 200 mW, and 300 and 350 mW where the area under the ROC curve is relatively lower. Finally, comparing 150 and 350 mW, the obtained ROC sufficiently shows an acceptable area under the curve (i.e., AUC > 0.7). In general, the obtained ROC curves show a good agreement with the experimental results.

The diffuse transmittance at skin samples shows high discrimination from 150 to 250 mW with high AUC values. Nonetheless, from 250 to 300 mW, the value is not significant which agree with the obtained scattering coefficient values. However, from 300 to 350 mW, the ROC curve restores its high AUC. The sensitivity, specificity, and accuracy for each ROC curve are summarized in Table 1.

**Table 1:** The obtained ROC curves parameters of skull and skin at different incident power.

Laser power	Sensitivity		Specificity		AUC		Accuracy	
	Skull	Skin	Skull	Skin	Skull	Skin	Skull	Skin
**150 and 200 mW**	0.82	0.98	0.77	0.98	0.86	0.98	0.79	0.98
**200 and 225mW**	0.98	0.91	0.98	0.90	0.98	0.95	0.98	0.90
**225 and 250 mW**	0.97	0.99	0.98	0.99	0.97	0.99	0.98	0.99
**250 and 300mW**	0.97	0.51	0.95	0.49	0.97	0.50	0.98	0.50
**300 and 350 mW**	0.82	0.87	0.77	0.86	0.86	0.93	0.79	0.86
**150 and 350 mW**	0.98	0.99	0.98	0.99	0.98	0.99	0.98	0.99

### Spatial fluence rate distribution

**Fig 6:**
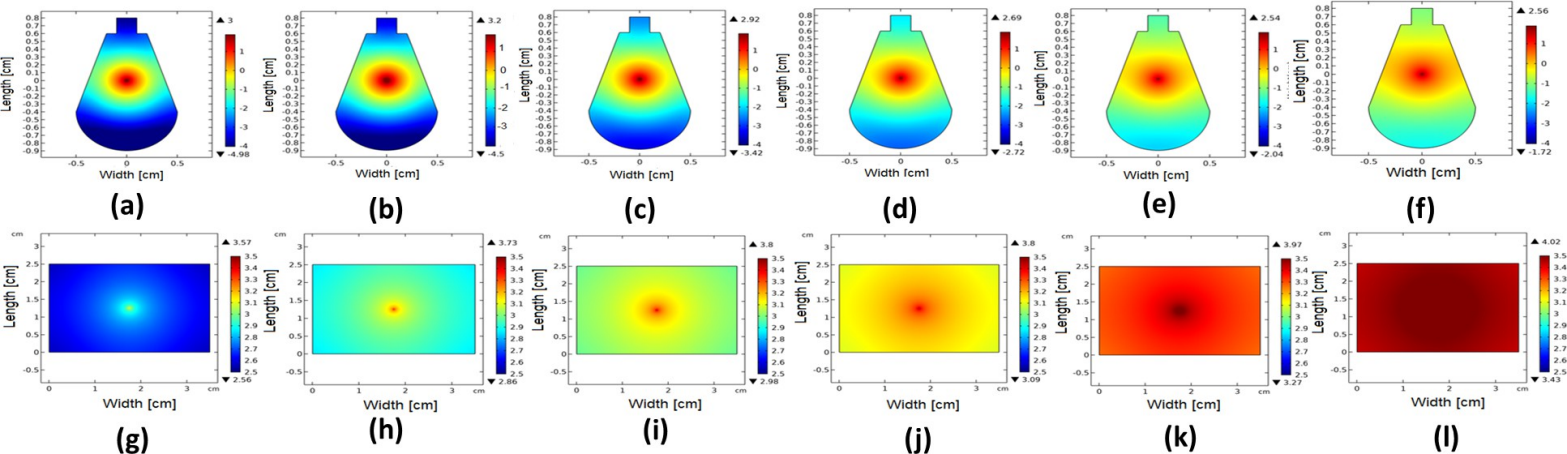
The estimated fluence rate distribution at the skull surface at (a) 150 mW, (b) 200 mW, (c) 225 mW, (d) 250 mW, (e) 300 mW, (f) 350 mW, and at the skin surface at (g) 150 mW (h) 200 mW (i) 225 mW (j) 250 mW (k) 300 mW (l) 350 mW.

The calculated optical absorption and scattering parameters for the examined samples have been introduced to the finite element model. The solution of diffusion equation is obtained to visualize the distribution of the fluence rate at the skull and skin surfaces at each incident laser power. The obtained results are presented in Fig 6.

As illustrated in Fig 6, the spatial fluence rate becomes more diffusive as the incident power increases at lower scattering coefficient values. The same behavior occurs at the skin surface, the distribution of the fluence at low incident power was more focused at the central region, while at higher incident power, it becomes more diffusive.

### Simulation of the laser propagation at different anisotropy factors

**Fig 7:**
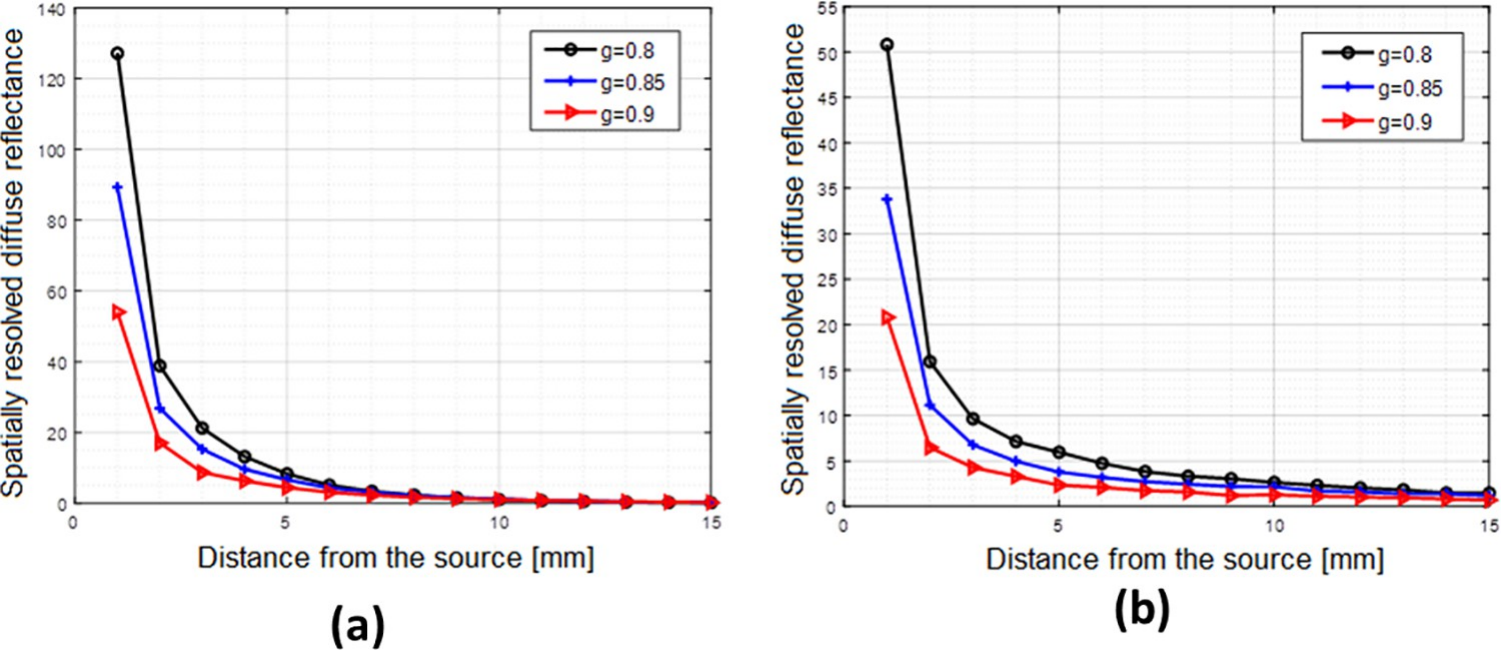
(a) Changing the spatial diffuse reflectance with the anisotropy factor in skull, (b) Changing the spatial diffuse reflectance with the anisotropy factor in skin.

The reduction of scattering characteristics by increasing the laser incident power has been verified via Monte-Carlo simulation. The diffuse reflectance, as a function of the spatial distance from the light source, for the examined skull and skin samples have been simulated at different anisotropy factors 0.8, 0.85 and 0.9, as presented in Fig 7.

As shown in Fig 7, the reflectance distribution shows lower values and becomes more attenuated at higher *g* values.

## Discussion

Investigating the variation of the optical parameters of some biological tissues with the laser light parameters is critical for obtaining the optimum outcome of diagnostics and treatment applications. Laser-tissue interaction has been the focus of several studies [43, 44] for and has been useful for the application of laser in a variety of applications such as hair removal [45], wound healing [46], and the treatment of some neurological diseases [47, 48]. Consequently, Investigating the change of optical parameters with laser power may restrict the use of laser in certain applications or may offer an adjustment for the safe use [49]. Therefore, the present investigation studies a crucial issue in the field due to its considerable effect on the experimental results.

During the last decades, an increasing number of transcutaneous and transcranial photobiostimulation applications have been emerged [50, 51]. Therefore, one of the most commonly used laser wavelength range (i.e., Infrared laser) in medical applications and the extensively studies tissue samples (skull and skin) have been utilized for the present investigation. In the present study, a decrease in the reduced scattering coefficient from 37 to 9 cm^-1^ and from 12 to 5.8 cm^-1^ for the skull and skin, respectively, in the range of incident laser power of 150 to 350 mW have been obtained. Theoretically, the hard tissue (skull) has a lower proclivity to change over time (e.g., drying out) than the soft tissue (skin) [52]. Despite this fact, the reduction of the scattering coefficient with the laser power for the skull is greater than that for the skin. This supports the proposed hypothesis that states that tissue scattering properties change with the incident IR laser power.

Optical scattering and absorption properties control the light propagation in biological tissues and greatly affect the required optical dose in different diagnostic and therapeutic applications. In a previous paper investigating the laser transmission profiles of the same wavelength as the present study through the rat’s head, we found that increasing the laser power has an effect on the tissue optical properties including scattering [53]. Laser light propagation encountered in biological structure experiences forward and multiple scattering due to the variation of the refractive indices and scattering particle sizes within the tissue layers. These phenomena are intimately correlated with the propagation of light in biological samples. Besides, light scattering in biological tissue depends on many factors related to its morphology, structure, and components such as cell membrane and organelles [5, 54].

Although precise measuring of diffuse reflectance is required for the determination of the optical parameters, tissue anisotropy factor ɡ is a very vital parameter that affects the calculation process of scattering coefficient [55]. The anisotropy factor represents the asymmetry of the single scattering event; it ranges from 0, 1, and -1 for extremely forward, isotropic, and highly backward scattering, respectively [56]. The connection between the reduced scattering coefficient and the scattering coefficient **μ**_**s**_ is the anisotropy factor ($\mu_{s}^{\prime }=\mu_{s}(1-g)$). That is, increasing *g* causes a decrease in $\mu_{s}^{\prime }$ which can result in a decrease in the reduced scattering coefficients presented on our results. It is known that, in the biological tissue, the forward scattering is highly dominating [57, 58] and correlated with the irradiation intensity and this, in turn, causes an elevation in the tissue anisotropy factor g. Additionally, increasing the anisotropy factor, by increasing the incident light intensity, has a great effect on both tissue reflectance and optical fluence rate distribution [59].

As reported in some relevant studies [60, 61], tissue diffuse reflectance increases as a result of increasing the scattering coefficient. The curves presented in Fig 7a, 7b demonstrate that tissue diffuse reflectance decreases at higher anisotropy factor which reveals a reduction of the scattering coefficient. The size and the index of refraction of the scatterers control the linear scattering cross-section. However, these parameters change upon increasing the intensity of the laser beam and, hence, affects the scattering cross-section [62]. This phenomenon affects the energy of the scattered light which, in turn, changes the optical properties of the studied medium [63]. In addition to diffuse reflectance and anisotropy factor, some studies reported that tissue absorption and scattering properties differ with tissue heating specially in the coagulation temperature range (from 40°C to about 80°C) [60, 61]. The thermal effect of the laser radiation is utilized to heat tissue fat and, hence, affects the optical scattering properties [64, 65].

Visualization of the distribution of the fluence rate at the skull and skin surfaces at each incident laser power, based on the scattering and absorption parameters, revealed that the more the power used the more diffusive the distribution of laser irradiation. Higher scattering at lower powers results in a distribution of more circular profile, and decreases the laser penetration. As the scattering is reduced by increasing incident laser power, the penetration depth is increased due to increasing the distance between the scattering events. Also, it results in a more diffusive pattern of distribution. This variation in scattering coefficient is closely related to the variation in the tissue anisotropy factor with incident laser power. Therefore, the laser distribution and penetration in tissue at different incident laser power are highly dependent on the variation of the two interrelated parameters; the tissue anisotropy and the scattering coefficient.

## Conclusion

A variation in the scattering of incident laser with the incident laser power has been investigated in the present study by experimental measurements and verified by Monte-Carlo simulation. The decrease in scattering has been observed in both skull and skin tissue samples with increasing the laser power. The penetration and fluence rate distribution has been also affected by varying the laser power. The present results comprise a controlled study in which this relationship has been established. The present findings should be taken into consideration when using a low-level laser either transcutaenously or transcranially for diagnosis and treatment purposes. Further investigations at different wavelengths and different tissues are needed to extrapolate the present findings.

## References

[1] Calin MA, Parasca SV, Savastru R, Calin MR, Dontu S. Optical techniques for the noninvasive diagnosis of skin cancer. J Cancer Res Clin Oncol. 2013. doi: 10.1007/s00432-013-1423-3 23552870 PMC11824606

[2] Lee K. Optical mammography: Diffuse optical imaging of breast cancer. World J Clin Oncol. 2011;2: 64–72. doi: 10.5306/wjco.v2.i1.64 21603315 PMC3095466

[3] Nachab´e R, Evers DJ, Hendriks BHW, Lucassen GW, Voort M van der, Rutgers EJ, et al. Diagnosis of breast cancer using diffuse optical spectroscopy from 500 to 1600 nm: comparison of classification methods. J Biomed Opt. 2011;16: 087010–1–12. doi: 10.1117/1.3611010 21895337

[4] O’Sullivan TD, Cerussi AE, Cuccia DJ, Tromberg BJ. Diffuse optical imaging using spatially and temporally modulated light. J Biomed Opt. 2012;17: 71311–1–14. doi: 10.1117/1.JBO.17.7.071311 22894472 PMC3607494

[5] Tuchin V V. Tissue optics: light scattering methods and instruments for medical diagnosis. Bellingham, Washington USA: SPIE PRESS; 2007. doi: 10.1117/3.684093

[6] Wang, Lihong V.; Wu H. Biomedical Optics: Principles and Imaging. Canada: Wiley-Interscience; 2007.

[7] Zhang Y, Chen Y, Yu Y, Xue X, Tuchin V V., Zhu D, et al. Visible and near-infrared spectroscopy for distinguishing malignant tumor tissue from benign tumor and normal breast tissues in vitro. J Biomed Opt. 2013;18: 077003–1–7. doi: 10.1117/1.JBO.18.7.077003 23839487

[8] Hamdy O, Abdelazeem RM. Toward Better Medical Diagnosis: Tissue Optical Clearing. J Public Heal Int. 2020;2: 13–21. doi: 10.14302/issn.2641

[9] Hamdy O, El-Azab J, Solouma NH, Fathy M, Al-Saeed TA. The use of optical fluence rate distribution for the differentiation of biological tissues. 8th Cairo International Biomedical Engineering Conference (CIBEC). 2016. doi: 10.1109/CIBEC.2016.7836129

[10] Hamdy O, Fathy M, Al-Saeed TA, El-Azab J, Solouma NH. Estimation of optical parameters and fluence rate distribution in biological tissues via a single integrating sphere optical setup. Optik. 2017;140: 1004–1009. doi: 10.1016/j.ijleo.2017.05.039

[11] Hamdy O, Abdel-Salam Z, Abdel-Harith M. Discrimination between fresh, chilled, and frozen/ thawed chicken based on its skin’s spectrochemical and optical properties. Anal Methods. 2020;12: 2093–2101. doi: 10.1039/d0ay00324g

[12] Hamdy O, Solouma NH. Distant-Detector versus Integrating Sphere Measurements for Estimating Tissue Optical Parameters: A Comparative Experimental Study. Optik (Stuttg). 2021;247: 167981. doi: 10.1016/j.ijleo.2021.167981

[13] Hamdy O, El-Azab J, Al-Saeed TA, Hassan MF, Solouma NH. A method for medical diagnosis based on optical fluence rate distribution at tissue surface. Materials (Basel). 2017;10: 1–13. doi: 10.3390/ma10091104 28930158 PMC5615757

[14] Abuelmakarem HS, Hamdy O, Sliem MA, El-azab J, M.A O-H, Ahmed WA. Colonic Carcinoma Diagnosis using Chitosan Nanoparticles Based on the Optical Properties. J Phys Conf Ser. 2020;1472: 012001–1–8. doi: 10.1088/1742-6596/1472/1/012001

[15] Mahdy S, Hamdy O, Hassan MA, Eldosoky MAA. A modified source ‑ detector configuration for the discrimination between normal and diseased human breast based on the continuous ‑ wave diffuse optical imaging approach: a simulation study. Lasers Med Sci. 2021. doi: 10.1007/s10103-021-03440-9 34651256

[16] M A, R B, Y L, SL J, K E, R P. Extraction of optical properties and prediction of light distribution in rat brain tissue. J Biomed Opt. 2014;19: 75001. doi: 10.1117/1.JBO.19.7.075001 24996660

[17] Firdous S, Ikram M, Nawaz M, Aslam M. Measurement of Optical Parameters: Absorption Scattering and Auto-florescence of Skin in vitro. Int J Cancer Res. 2005;1: 10–15.

[18] Prahl SA, van Gemert MJ, Welch AJ. Determining the optical properties of turbid mediaby using the adding-doubling method. Appl Opt. 1993;32: 559–68. doi: 10.1364/AO.32.000559 20802725

[19] Bashkatov AN, Genina EA, Tuchin V V. Optical Properties of Skin, Subcutaneous, and Muscle Tissues: A Review. J Innov Opt Health Sci. 2011;4: 9–38. doi: 10.1142/S1793545811001319

[20] Soleimanzad H, Pain F. Optical properties of mice skull bone in the 455- to 705-nm range skull bone in the 455- to 705-nm range. J Biomed Opt. 2017;22: 010503–1–4. doi: 10.1117/1.JBO.22.4.049802 28399197

[21] Ishii K, Kimura A, Awazu K. Optical properties of tissues after laser treatments in the wavelength range of 350–1000 nm. Proc SPIE. 2008;6991: 69912F. doi: 10.1117/12.781607

[22] Kim S, Jeong S. Effects of temperature-dependent optical properties on the fluence rate and temperature of biological tissue during low-level laser therapy. Lasers Med Sci. 2014;29: 637–644. doi: 10.1007/s10103-013-1376-4 23807181

[23] Genina EA, Bashkatov AN, Sinichkin YP, Yanina IY, Tuchin V V. Optical clearing of biological tissues: prospects of application in medical diagnostics and phototherapy. J Biomed Photonics Eng. 2015;1: 23–58.

[24] Dompe C, Moncrieff L, Matys J, Grzech-Leśniak K, Kocherova I, Bryja A, et al. Photobiomodulation—Underlying Mechanism and Clinical Applications. J Clin Med. 2020;9: 1–17. doi: 10.3390/jcm9061724 32503238 PMC7356229

[25] Markolf NH. Laser-Tissue Interactions: Fundamentals and Applications. Germany: Springer; 2007.

[26] Wei H-J, Xing D, Wu G-Y, Jin Y, Gu H-M. Optical properties of human normal small intestine tissue determined by Kubelka-Munk method in vitro. World J Gastroenterol. 2003;9: 2068–2072. doi: 10.3748/wjg.v9.i9.2068 12970908 PMC4656676

[27] Boas DA, Pitris C, Ramanujam N. Handbook of Biomedical Optics. CRC press; 2011.

[28] Kottler F. Turbid Media with Plane-Parallel Surfaces. J Opt Soc Am. 1960;50: 483. doi: 10.1364/josa.50.000483

[29] Manuel L, Oliveira C. The Optical Clearing Method A New Tool for Clinical Practice and Biomedical Engineering. Springer; 2019.

[30] Sterenborg HJCM, Veen RLP van, Aans J-B, Amelink A, Robinson DJ. Light dosimetry for photodynamic therapy: Basic concepts. Handbook of Photomedicine. CRC Press; 2013.

[31] Vining DJ, Gladisb GW. Receiver Operating Characteristic Curves: A Basic Understanding. Radiographics. 1992;12: 1147–1154. doi: 10.1148/radiographics.12.6.1439017 1439017

[32] Hajian-Tilaki K. Receiver Operating Characteristic (ROC) Curve Analysis for Medical Diagnostic Test Evaluation. Casp J Intern Med. 2013;4: 627–635. 24009950 PMC3755824

[33] Streiner DL, Cairney J. What’s Under the ROC? An Introduction to Receiver Operating Characteristics Curves. Res Methods Psychiatry. 2007;52: 121–128. doi: 10.1177/070674370705200210 17375868

[34] Nam B-H, D’Agostino RB. Discrimination Index, the Area Under the ROC Curve. Statistics for Industry and Technology. Birkhäuser, Boston, MA; 2002. pp. 267–279. doi: 10.1007/978-1-4612-0103-8_20

[35] Goncalves L, Subtil A, Oliveira MR, Bermudez P de Z. ROC Curve Estimation: An Overview. REVSTAT–Stat J. 2014;12: 1–20.

[36] Zou KH, O’Malley AJ, Mauri L. Receiver-Operating Characteristic Analysis for Evaluating Diagnostic Tests and Predictive Models. Circulation. 2007;115: 654–657. doi: 10.1161/CIRCULATIONAHA.105.594929 17283280

[37] Zhu C, Liu Q. Review of Monte Carlo modeling of light transport in tissues Review of Monte Carlo modeling of light transport. J Biomed Opt. 2013;18: 050902–1–12. doi: 10.1117/1.JBO.18.5.050902 23698318

[38] Key H, Davies ER, Jackson PC, Wells PNT. Monte Carlo modelling of light propagation in breast tissue. Phys Med Biol. 1991;36: 591–602. doi: 10.1088/0031-9155/36/5/003 2068225

[39] Atif M, Khan A, Ikram M. Modeling of Light Propagation in Turbid Medium Using Monte Carlo Simulation Technique. Opt Spectrosc. 2011;111: 107–112. doi: 10.1134/S0030400X11070022

[40] Wang L, Jacquesa SL, Zhengb L. MCML—Monte Carlo modeling of light transport in. Comput Methods Programs Biomed 47. 1995;47: 131–146. doi: 10.1016/0169-2607(95)01640-f 7587160

[41] Hamdy O, Ismail T. Study of Optical Power Variations in Multi-layer Human Skin Model for Monitoring the Light Dose. Novel Intelligent and Leading Emerging Sciences Conference (NILES). 2019. pp. 21–24. doi: 10.1109/NILES.2019.8909332

[42] Hamdy O, Youssef D, El-azab J. Detection of Breast Diseases using Numerical Study of Light Propagation. 2018 9th Cairo Int Biomed Eng Conf. 2018; 53–56.

[43] Bordin-Aykroyd S, Dias RB, Lynch E. Laser-Tissue Interaction. EC Dent Sci. 2019;18: 2303–2308.

[44] Ash C, Dubec M, Donne K, Bashford T. Effect of wavelength and beam width on penetration in light-tissue interaction using computational methods. Lasers Med Sci. 2017;32: 1909–1918. doi: 10.1007/s10103-017-2317-4 28900751 PMC5653719

[45] Lepselter J, Elman M. Biological and clinical aspects in laser hair removal. J Dermatolog Treat. 2004;15: 72–83. doi: 10.1080/09546630310023152 15204156

[46] Samaneh R, Ali Y, Mostafa J, Mahmud NA, Zohre R. Laser Therapy for Wound Healing: A Review of Current Techniques and Mechanisms of Action. Biosci Biotechnol Res Asia. 2015;12: 217–223.

[47] Siposan D. Lasers in neurology. Lasers for medical applications: Diagnostics, therapy and surgery. Woodhead Publishing Limited; 2013. pp. 573–603. doi: 10.1533/9780857097545.4.573

[48] Mohammed HS. Transcranial low-level infrared laser irradiation ameliorates depression induced by reserpine in rats. Lasers Med Sci. 2016;31: 1651–1656. doi: 10.1007/s10103-016-2033-5 27437987

[49] Barbora A, Bohar O, Sivan AA, Magory E, Nause A, Minnes R. Higher pulse frequency of near-infrared laser irradiation increases penetration depth for novel biomedical applications. PLoS One. 2021;16: e0245350. doi: 10.1371/journal.pone.0245350 33411831 PMC7790424

[50] Huang Y-Y, Chen AC-H, Carroll JD, Hamblin MR. Biphasic Dose Response in Low Level Light Therapy. Dose-Response. 2009. doi: 10.2203/dose-response.09-027.Hamblin 20011653 PMC2790317

[51] Chang J, Ren Y, Wang R, Li C, Wang Y, Chu X. Transcranial Low-Level Laser Therapy for Depression and Alzheimer ‘ s Disease. Neuropsychiatry (London). 2018;8: 477–483.

[52] Wilkinson C. Facial reconstruction—anatomical art or artistic anatomy? J Anat. 2010;216: 235–250. doi: 10.1111/j.1469-7580.2009.01182.x 20447245 PMC2815945

[53] Hamdy O, Mohammed HS. Investigating the transmission profiles of 808 nm laser through different regions of the rat ‘ s head. Lasers Med Sci. 2021;36: 803–810. doi: 10.1007/s10103-020-03098-9 32638241

[54] Niemz MH. Laser-Tissue Interactions: Fundamentals and Applications. Fourth. Springer Nature Switzerland; 2019.

[55] Fukutomi D, Ishii K, Awazu K. Determination of the scattering coefficient of biological tissue considering the wavelength and absorption dependence of the anisotropy factor. Opt Rev. 2016;23: 291–298. doi: 10.1007/s10043-015-0161-y

[56] Cheong W-F, Prahl SA, Welch AJ. A Review of the Optical Properties of Biological Tissues. IEEE J Quantum Electron. 1990;26: 2166–2185.

[57] Tuchin V V., Utz SR, Yaroslavsky llya V. Tissue optics, light distribution, and spectroscopy. Opt Eng. 1994;33: 3178–3188. doi: 10.1117/12.178900

[58] Wang Lihong V.; Wu H. Biomedical Optics: Principles and Imaging. Canada: WILEY-INTERSCIENCE; 2007.

[59] Priyanka ND, Kumar A. Effect of Anisotropy and Scattering Coefficient on Light Distribution in a Semi-infinite Tissue. International Conference on Modeling and Simulation of Diffusive Processes and Applications. 2012.

[60] Qilesiz IF, Welch AJ. Light dosimetry: effects of dehydration and thermal damage on the optical properties of the human aorta. Appl Opt. 1993;32: 477–487. doi: 10.1364/AO.32.000477 20802715

[61] Jaywant S, Wilson B, Patterson M, Lilge L, Flott T., Woolsey J, et al. Temperature dependent changes in the optical absorption and scattering spectra of tissues. SPIE. 1993;1882: 218–229. doi: 10.1117/12.148080

[62] Vanyukov V. Effects of nonlinear light scattering on optical limiting in nanocarbon suspensions. The University of Eastern Finland. 2015. Available: http://urn.fi/URN:ISBN:978-952-61-1824-6.

[63] Striebel M, Wrachtrup J, Gerhardt I. Absorption and Extinction Cross Sections and Photon Streamlines in the Optical Near-field. Sci Rep. 2017;7: 1–13. doi: 10.1038/s41598-016-0028-x 29133925 PMC5684246

[64] Tuchin V V., Yanina IY, Simonenko G V. Destructive fat tissue engineering using photodynamic and selective photothermal effects. Proc SPIE 7179, Optics in Tissue Engineering and Regenerative Medicine III. 2009. doi: 10.1117/12.812164

[65] Dubrovskii VA, Dvorkin BA, Yanina IY, Tuchin V V. Photoaction upon Adipose Tissue Cells in vitro. Cell tissue biol. 2011;5: 520–529. doi: 10.1134/S1990519X1105004X 21786686

